# Effectiveness of Dual Sympathetic Blocks for Sympathetically Mediated Symptoms in Post-acute Sequelae of SARS-CoV-2 (PASC): An Open-Label, Non-randomized Pilot Study

**DOI:** 10.7759/cureus.81530

**Published:** 2025-03-31

**Authors:** Serena Wang, Richard J Salway, Megan Nicklay, Jonathann Kuo

**Affiliations:** 1 Pain Management, Hudson Medical Group, New York, USA; 2 Emergency Medicine, Extension Health, New York, USA; 3 Pain Management, Extension Health, New York, USA

**Keywords:** autonomic dysfunction, cardiovascular autonomic dysfunction (cvad), cerebral blood flow (cbf), dual sympathetic blocks, long covid, pasc (post-acute sequelae of sars-cov-2), pcc (post-covid conditions), stellate ganglion block

## Abstract

Background: Post-acute sequelae of SARS-CoV-2 (PASC), or Long COVID, is a multi-system disorder often involving dysautonomia and pain, linked to persistent sympathetic nervous system hyperactivity. Dual sympathetic blocks (DSBs), including stellate ganglion blocks (SGBs), are minimally invasive procedures that may recalibrate dysregulated sympathetic signaling and alleviate PASC symptoms.

Methods: This open-label, non-randomized pilot study included 20 participants with PASC experiencing pain and autonomic symptoms. Participants received right-sided and left-sided SGB procedures one week apart. Symptoms were assessed at baseline, week one, and week four using the Composite Autonomic Symptom Score (COMPASS-31) and Patient-Reported Outcomes Measurement Information System (PROMIS-29) scales.

Results: Seventeen participants completed the study, demonstrating significant improvements in autonomic dysfunction (38.4% reduction in COMPASS-31 scores, p = 0.0016) and pain interference (48.4% reduction, p < 0.001) by week four. Improvements in sleep quality and fatigue were also significant by week four (p = 0.016 and p = 0.049, respectively). Overall, 88.2% of participants reported symptom relief, and no adverse events were observed.

Conclusions: DSBs appear to be a promising intervention for PASC-related dysautonomia and pain. These findings warrant further investigation in larger, randomized controlled trials to confirm efficacy and explore the underlying mechanisms.

## Introduction

Post-acute sequelae of SARS-CoV-2

Since the emergence of the COVID-19 pandemic, the necessity for swiftly advancing research initiatives and developing treatment protocols has continued to escalate. Although some individuals recover rapidly from COVID-19, a growing number of people who were previously infected with the SARS-CoV-2 virus have reported experiencing persistent, recurring, or newly arising health complications well after their initial recovery. The Centers for Disease Control and Prevention (CDC) and the National Institutes of Health (NIH) classify these prolonged effects of COVID-19 under the comprehensive research term post-acute sequelae of SARS-CoV-2 (PASC), which is defined by the presence of continuing or returning symptoms in individuals who have recovered from an acute SARS-CoV-2 infection [1].

PASC, which is interchangeably referred to as post-COVID conditions (PCCs) or Long COVID, manifests in various ways and can impair the function of multiple organ systems, including the respiratory, neurological, and digestive systems. The most frequently observed symptoms of PASC include fatigue, post-exertional malaise, shortness of breath, cognitive impairment commonly known as “brain fog,” sleep disturbances, fever, anxiety, and depression [2]. Additional symptoms reported by PASC patients consist of persistent cough, chest pain or discomfort, headaches, heart palpitations, muscle or joint pain, nausea, abdominal pain, dizziness, diarrhea, anosmia (loss of smell), or ageusia (loss of taste) [2]. Symptom types differ between patients, and severity also varies from patient to patient.

Currently, no definitive test exists to diagnose PCCs. The symptoms associated with PASC are often non-specific, and standard clinical evaluations-including blood tests, chest X-rays, and electrocardiograms-may return normal results, making diagnosis particularly challenging for healthcare providers. PASC is more common in individuals who had severe COVID-19, but even those with mild or asymptomatic infections can develop it. Physicians typically diagnose PCCs based on a patient’s medical history, taking into account whether they previously had a confirmed COVID-19 diagnosis through testing, symptoms, or known exposure to the virus [1]. The impact of PASC extends beyond physical symptoms, often interfering with daily life, work, and education. Many patients report that Long COVID symptoms prevent them from resuming their jobs or studies and significantly impair their ability to complete routine tasks, such as walking short distances. Pain management centers treating PASC patients indicate that a majority of cases meet the criteria for chronic primary pain with major distress, as outlined in the ICD-11 chapter on chronic pain [3,4]. Furthermore, recent legal guidance has affirmed that PASC, or Long COVID, may qualify as a disability under the Americans with Disabilities Act (ADA) if it significantly restricts one or more major life activities.

Ongoing research and growing scientific understanding of PASC suggest that it closely resembles certain chronic pain syndromes and conditions characterized by sympathetic nervous system dysfunction, both of which may be responsive to existing therapeutic interventions [5-9]. Given that the symptoms can be highly debilitating and lead to profound reductions in quality of life, developing effective treatment and management strategies for this condition remains a critical priority in post-pandemic healthcare. Identifying targeted therapeutic approaches will be essential in addressing the widespread implications of PASC in the aftermath of the COVID-19 pandemic.

Dysautonomia in PASC

Since PASC has only recently been recognized as a distinct disease entity, its underlying mechanisms remain largely unclear, and currently, there are limited targeted and effective treatment options available. As PASC can impact multiple organ systems, treatment approaches are typically multi-disciplinary, aiming to manage symptoms while addressing underlying health issues [1]. A significant number of PASC symptoms that do not respond adequately to conventional treatment approaches are linked to dysautonomia [5,7,9,10]. Dysautonomia refers to the dysfunction of involuntary physiological processes, primarily regulated by the sympathetic nervous system, including heart rate, respiration, and digestion [10]. The sympathetic nervous system plays a crucial role in the interaction between the immune system and nervous system, but disruptions-such as increased cytokine levels-can impair this connection [11,12].

The well-documented cytokine storm seen in SARS-CoV-2 infections directly results from autonomic system activation, wherein the sympathetic nervous system overreacts to pro-inflammatory cytokines [13]. When sympathetic activity remains elevated, the brainstem integrates these signals, leading to sickness behaviors, which closely mirror the symptoms observed in PASC [14]. Given that PASC symptoms can persist for weeks or even months, prolonged excessive sympathetic signaling could potentially worsen the overall condition.

Recent research also suggests that autonomic dysfunction in Long COVID may result from neuroinflammation, persistent immune activation, and cytokine-related damage to autonomic pathways. This inflammation likely stems from direct viral invasion of the nervous system and disruption of the blood-brain barrier. Persistent immune cell activation has been linked to damage in autonomic control centers in the brainstem and peripheral nervous system [15]. These processes contribute to autonomic instability, leading to symptoms like dizziness, fatigue, and irregular cardiovascular responses. Additionally, residual viral antigens and similarities between viral proteins and host tissues may trigger an ongoing autoimmune reaction, further worsening autonomic dysfunction in affected individuals.

Additionally, chronic dysautonomia is closely linked to impaired cerebral blood flow (CBF) in conditions such as myalgic encephalomyelitis/chronic fatigue syndrome (ME/CFS), postural orthostatic tachycardia syndrome (POTS), and other chronic illnesses associated with sympathetic nervous system dysfunction [8,14,16,17]. Reduced CBF is known to cause a range of neurological symptoms, such as cognitive impairment, memory deficits, attention difficulties, and diminished sensory processing, including vision, taste, and smell [8]. Recent studies have also identified that cardiovascular autonomic dysfunction (CVAD) is a significant component of post-COVID-19 syndrome, affecting approximately one-third of highly symptomatic Long COVID patients [18]. CVAD presents as impairments in heart rate and blood pressure regulation, often manifesting as POTS, inappropriate sinus tachycardia (IST), and microvascular dysfunction. The symptom profile of these conditions aligns with many of the symptoms observed in PASC patients. As research continues, ongoing investigations and clinical observations seek to further clarify the pathophysiology of PASC and identify more effective treatment strategies.

A 2021 case series provided preliminary evidence supporting stellate ganglion blocks (SGBs) as a potential intervention for alleviating Long COVID or PASC symptoms. The study documented sustained clinical improvement in two Long COVID patients following SGB treatment, indicating that their symptoms were driven by sympathetically mediated dysautonomia [14]. The findings suggest that SGB could serve as a viable therapeutic option for certain PASC patients. Although the application of SGB in PASC treatment is still considered novel, researchers highlight its potential as an appealing and promising therapy for a condition that currently lacks effective treatment solutions.

Dual sympathetic blocks (SGBs)

The stellate ganglion serves as a crucial nerve center that facilitates communication between the central and peripheral nervous systems. By blocking this neural pathway, it may be possible to recalibrate dysregulated sympathetic signaling, which in turn could help reduce inflammation, enhance CBF, and restore autonomic balance [16]. Sympathetic nerve blocks are well-established interventional procedures utilized by many pain management specialists as a reliable approach for both diagnosing and treating pain conditions related to sympathetic nervous system dysfunction [17,19].

For decades, the stellate ganglion nerve block has been used as a treatment for complex, sympathetically mediated pain syndromes that affect the head, face, neck, and arms [20]. This nerve bundle transmits sympathetic signals to various parts of the body, including the head, neck, upper limbs, thymus, heart, lungs, lacrimal gland, salivary gland, thyroid gland, and pineal gland. When a local anesthetic is injected near the stellate ganglion, it effectively disrupts sympathetic activity along the entire cervical sympathetic chain [14]. The success of an SGB is often confirmed by the onset of Horner’s syndrome, a collection of physiological signs that include ipsilateral ptosis (drooping eyelid), meiosis (constricted pupil), anhidrosis (reduced sweating), and facial flushing [21].

Research suggests that SGB may help alleviate dysautonomia symptoms by facilitating regional recalibration of sympathetic influence, promoting central integration of improved CBF, and rebalancing interactions between the nervous and immune systems [14]. Although PASC has only recently been defined as a distinct disease entity, growing research continues to support the theory that SARS-CoV-2 infections impact the autonomic nervous system (ANS). Increasing evidence indicates that PASC shares key characteristics with many chronic pain syndromes, and dysautonomia appears to play a central role in both acute and chronic phases of SARS-CoV-2 infection [5-7]. Previous studies have demonstrated that similar autonomic recalibration mechanisms provide symptom relief in other conditions, reinforcing the potential application of dual sympathetic blocks (DSBs) for treating PASC-related dysautonomia [21]. Therefore, sympathetic nerve blocks, including SGB, align with existing clinical indications for treatments aimed at diagnosing and managing pain conditions that involve the sympathetic nervous system, further underscoring their relevance in addressing PASC-associated autonomic dysfunction.

This pilot study aimed to evaluate whether DSBs, including SGBs, can improve symptoms in patients with PASC, particularly those related to autonomic dysfunction and pain. Building on emerging evidence that links Long COVID symptoms to sympathetic nervous system dysregulation, we assessed changes in symptom severity using validated measures (Composite Autonomic Symptom Score (COMPASS-31) and Patient-Reported Outcomes Measurement Information System (PROMIS-29)) before and after treatment. We hypothesized that DSB would lead to meaningful reductions in autonomic symptoms and pain interference following both right- and left-sided procedures.

## Materials and methods

Participants

A total of 20 adults were enrolled in this pilot study, with 17 completing the study (four men and 13 women; age range 26-69). Participants were recruited through the principal investigator's pain management practice, local post-COVID care clinics, and promotional efforts, including Instagram (Meta Platforms, Inc., Menlo Park, CA, US) advertisements (@hudsonmedical) targeting individuals aged 18 and older. The inclusion criteria required participants to have a prior confirmed COVID-19 diagnosis, persistent symptoms continuing at least four weeks post-infection, quantified autonomic symptoms on COMPASS-31, and quantified pain symptoms of pain interference > 4 or pain intensity > 0 on PROMIS-29. Interested participants underwent Health Insurance Portability and Accountability Act (HIPAA)-compliant screening before enrollment.

Study design

This is a prospective, open-label, non-randomized pilot study evaluating the effectiveness of DSBs for patients experiencing sympathetically mediated symptoms from PASC. This study was approved by the Biomedical Research Alliance of New York (BRANY) #22-02-733-993 (Investigator Initiated Protocol #2022-250) and registered with ClinicalTrials.gov (NCT05638620). Twenty adult participants with PASC were enrolled in the study, which was held at a single center (Hudson Health/Extension Health). All subjects had a history of a prior confirmed COVID-19 diagnosis and met the current criteria for PASC including symptoms of pain that interferes with daily function and at least one autonomic symptom that recurs or persists for four weeks or more after an acute SARS-CoV-2 infection (Table 1). All participants provided written informed consent prior to participation.

**Table 1:** Inclusion and Exclusion Criteria

Inclusion criteria	Exclusion criteria
Age 18 or older	Comorbid conditions that could confound study results, such as active autoimmune disorders, pre-existing autonomic dysfunction (e.g., POTS and CRPS) prior to COVID-19, or untreated psychiatric conditions (e.g., severe anxiety or PTSD requiring medication adjustments during the study period)
Prior confirmed COVID-19 diagnosis (diagnostic or antibody test)	Use of medications that significantly affect autonomic function, including beta-blockers, midodrine, or other autonomic-modulating drugs within four weeks of enrollment
Persistent symptoms lasting at least four weeks post-infection	Recent history of major surgery, myocardial infarction, or cerebrovascular events within three months of study participation
Presence of autonomic dysfunction, as quantified by a Composite Autonomic Symptom Score (COMPASS-31)	Contraindications to stellate ganglion block (SGB), including active infection at the injection site, bleeding disorders, current anticoagulation therapy, or known allergies to local anesthetics
Presence of pain symptoms, defined as pain interference > 4 or pain intensity > 0 on the Patient-Reported Outcomes Measurement Information System (PROMIS-29)	

Patients were evaluated for pain interference and dysautonomic symptomatology by the PROMIS-29 and COMPASS-31 scores at zero, one, and four weeks. Study intervention will be administered at week zero and week one. The week four follow-up visit that will be used to assess the treatment intervention outcome will be either an in-office visit or virtual telehealth, depending on the patient’s preference. The study involves a total of three visits (week zero, week one, and week four) spanning the course of one month.

On the day of the procedure (week zero and week one), clinical staff performed standard intake to include a brief interim history, review of systems, vital signs, and placement of an intravenous catheter. The attending physician performed a targeted history and physical, paying attention to potential contraindications to SGB (e.g., infection at the site of injection, current anticoagulated state, presence of mass distorting the tissues, recent myocardial infarction, contralateral phrenic nerve palsy, and glaucoma). The physician will also give a brief explanation of the procedure as well as a review of risks and potential benefits, though these will have been described to the participants beforehand. Injections will be performed under ultrasound visualization. Immediately following the procedure, the participant was observed in the procedure operating room before transport to the recovery area for assessment of potential complications that could require immediate intervention, according to local clinic policy. In the post-procedure recovery area, monitoring of vital signs will continue for 20 minutes or longer, as dictated by clinic policy and participant condition. The clinical staff will use metrics to assess Horner's syndrome and recognize a successful block.

Procedure

**Figure 1:**
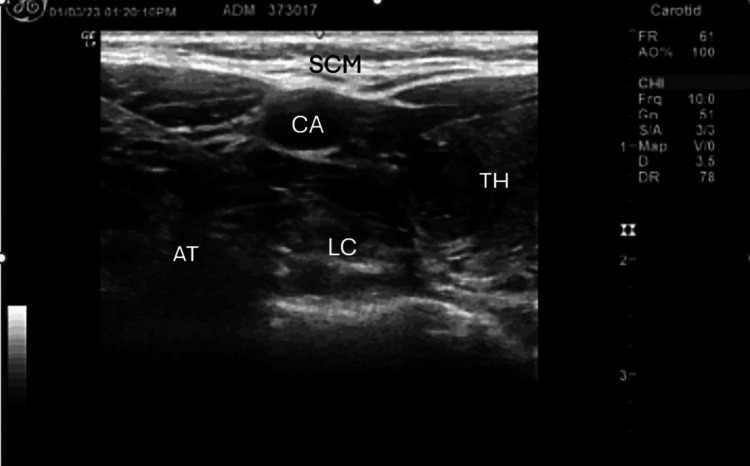
Ultrasound Image of SGB Injection SGB: stellate ganglion block; CA: carotid artery; LC: longus colli muscle; AT: anterior tubercle; TH: thyroid; SCM: sternocleidomastoid muscle

DSBs of the stellate ganglion are minimally invasive outpatient procedures performed under monitored care anesthesia (light sedation). Patients undergo a pre-injection medical history and physical examination. Using our site protocol, under ultrasound visualization, a gauge is guided into the neck region that contains the stellate ganglion nerve cluster at C6-C7 (Figure 1). Once the needle position is confirmed, a local anesthetic (7 cc of 0.5% bupivacaine/Marcaine) is injected around the stellate ganglion by the principal investigator. This procedure is repeated at the C3-C4 level to block the superior cervical ganglion nerve cluster (3 cc of 0.5% bupivacaine/Marcaine). The average procedure time is 20 minutes. Bupivacaine/Marcaine is FDA-approved for the production of local or regional anesthesia for surgery and acute pain management, including in the head and neck area.

The SGB is done on both sides of the neck. The SGB will be performed on the right side at the first visit. The procedure will be repeated on the left side one week after the first injection.

Outcome measures

Self-report assessments were made at three time points: pre-intervention (zero weeks), post right-sided SGB (one week), and post left-sided SGB (four weeks). Additional monitoring includes physical examinations performed during screening at visit one and all subsequent visits. The investigator examined the participants, and all participants had their blood pressure and oxygen saturation recorded. Vital signs were recorded during visits one, two, and three. Physical exams and vital signs were performed according to standard site protocols. These were recorded only in subjects' medical records and used in the case of reportable side effects.

Assessments

This study utilizes two assessment tools to monitor dysautonomic and overall function in patients with PASC. The COMPASS-31 questionnaire is a widely validated tool to assess symptoms of ANS dysfunction, and it evaluates six domains related to ANS function: orthostatic intolerance, vasomotor, secretomotor, gastrointestinal, urinary, and pupillomotor. To better assess post-COVID autonomic dysfunction, the questions included in this study’s surveys refer to the period after initial SARS-CoV-2 infection, and evaluation of changes in symptoms at each follow-up will be compared to when the dysautonomic symptoms appeared post-COVID [9,22]. Additionally, the impact of post-COVID symptoms on patients’ overall function and quality of life will be assessed by utilizing PROMIS-29, a validated set of person-centered measures to evaluate and monitor physical, mental, and social health [23].

Data analysis

Data was collected from January 3, 2023, until June 15, 2023. Seventeen out of the 20 participants enrolled completed the study. Among the 17 patients, 16 patients completed all necessary follow-up visits and surveys. One patient did not complete the PROMIS-29 survey at the week four follow-up visit and was excluded from the data analysis. Mean values for each domain score of the PROMIS-29 and the COMPASS-31 were calculated at baseline and multiple times post-intervention. This analysis will assess changes in autonomic and pain symptoms over time.

Upon finding significance, two-tailed paired t-tests, with a p-value of <0.05, were used to evaluate changes in mean scores at each time point and compared to baseline overall COMPASS-31 score and the PROMIS-29 subscales, including pain interference, pain intensity, fatigue, and sleep disturbance. Descriptive statistics will be used to summarize baseline characteristics, including age, sex, and symptom profiles.

## Results

**Figure 2:**
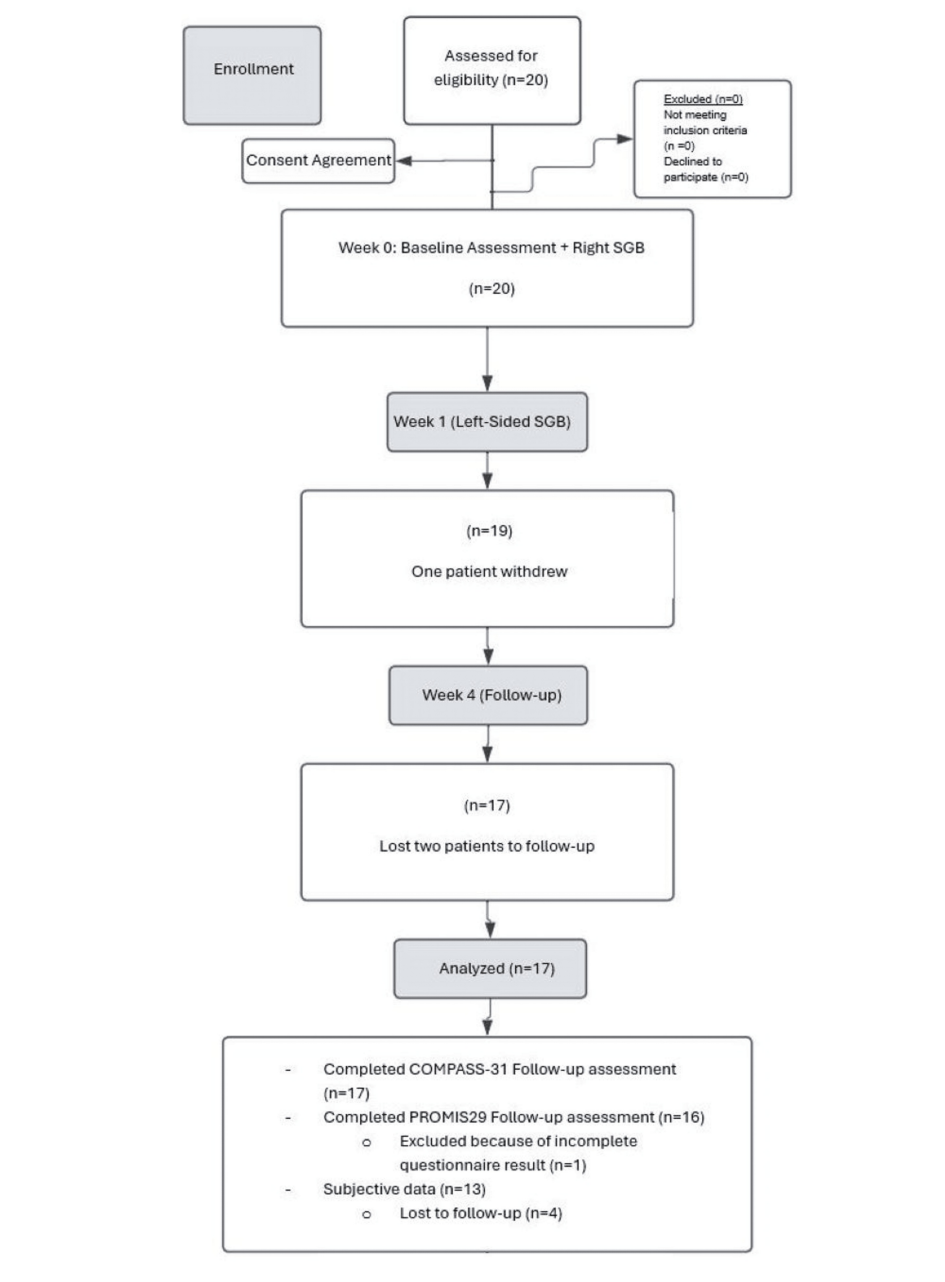
CONSORT Flow Diagram CONSORT: Consolidated Standards of Reporting Trials

Participants (Table 2) were tested at three time points: pre-intervention (zero weeks), post right-sided SGB (one week), and post left-sided SGB (four weeks) (Figure 2). The primary outcome was improvement in overall autonomic symptoms, orthostatic intolerance, pain interference, and pain scale. Secondary outcomes included less fatigue and better quality of sleep.

**Table 2:** Participant Demographics

Variable	Total
Male	6 (30%)
Female	14 (70%)
Age (18-29)	1 (5%)
Age (30-39)	10 (50%)
Age (40-49)	5 (25%)
Age (50-59)	3 (15%)
Age (60-69)	1 (5%)
Age (70+)	0 (0%)
Vaccinated	12 (60%)
Not vaccinated	8 (40%)
Hospitalized	0 (0%)
Not hospitalized	20 (100%)

Primary outcomes

**Figure 3:**
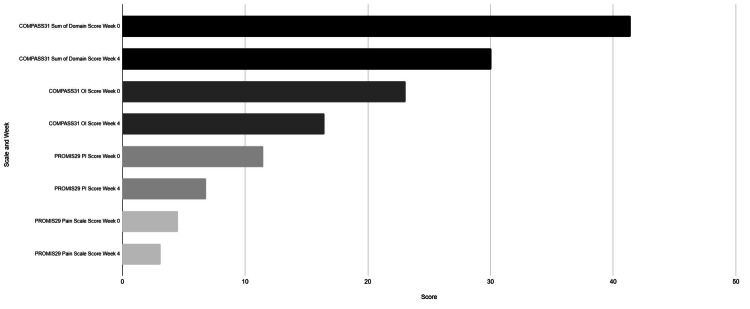
COMPASS-31 and PROMIS-29 Scores at Week 0 vs. Week 4

At the four-week follow-up, 13 of 17 participants (76.5%) demonstrated improvement in autonomic symptoms, as assessed by the COMPASS-31 scale. The mean overall COMPASS-31 score decreased by 38.4% (median: 34.5%), reflecting a significant reduction in symptom burden (Figure 3). The most notable improvements were observed in the orthostatic intolerance domain, which includes symptoms like dizziness, lightheadedness, tachycardia, and fatigue upon standing. Nine out of 17 patients demonstrated improvement at the four-week time point compared to baseline, with an average decrease of 44.7% in orthostatic intolerance symptoms (Figure 3). Paired t-test analysis demonstrated a statistically significant reduction in total COMPASS-31 scores from baseline to week four (p = 0.0016). Improvements in the average orthostatic intolerance score were significant at week four (p = 0.012) but did not reach significance at week one (p = 0.089), suggesting a delayed but meaningful therapeutic effect of the SGB treatment.

Pain interference, defined as the extent to which pain disrupts daily activities, showed the most significant improvement among the PROMIS-29 domains. A total of 13 out of 16 participants (81.3%) experienced a reduction in pain interference, with an average decrease of 48.4% (median: 50%) (Figure 3). Paired t-tests revealed significant improvements at both week one (p = 0.01) and week four (p = 0.00073), indicating both early and sustained symptom relief.

Similarly, pain intensity scores demonstrated significant reductions, with improvements noted as early as week one (p = 0.006) and persisting through week four (p = 0.009). The average of 13 out of 16 participants demonstrated a decrease in pain by 29.9% after the first SGB and a decrease of 42.6% after the second SGB (Figure 3). These findings suggest that SGB not only alleviates pain-related disability but also reduces overall pain perception over time.

Secondary outcomes

Secondary outcomes included improvements in sleep and fatigue. The PROMIS-29 fatigue scores showed marginal improvement at week one (p = 0.052) and significant improvement by week four (p = 0.049). By week one, eight out of 16 patients noticed less fatigue (M = 20.8%) and continued improvement after the second SGB at week four (M = 30.7%). Similarly, the PROMIS-29 sleep disturbance scores demonstrated significant improvement both at week one (p = 0.025) and week four (p = 0.016). Ten out of the 16 patients who noted improvement in sleep had an average of 22.2% less sleep disturbance by week one and 29.9% by week four. Furthermore, 56% of patients noticed an improvement in their anxiety symptoms, and 38% of patients noticed an improvement in their depression symptoms after both SGB treatments.

Subjectively, 15 of 17 participants (88.2%) reported improvements in their Long COVID symptoms after one or two SGB treatments, while two patients (11.8%) noted no significant changes. Patients who noted improvement in their symptoms mentioned less dizziness, decreased heart rate, improved sleep, more energy, improved functional mobility, less brain fog and anxiety, and improvement in temperature regulation. No participants reported worsening symptoms following the intervention.

## Discussion

This pilot study provides preliminary evidence that DSBs may offer a promising minimally invasive approach to alleviating sympathetically mediated symptoms in patients with PASC. We observed significant reductions in autonomic dysfunction, pain interference, and pain intensity following the administration of DSB, with improvements noted as early as one week and sustained at four weeks post-intervention. The observed reductions in pain interference and intensity may reflect not only the direct effects of local anesthetic but also its influence on pain processing pathways within the central nervous system [16,17,19,20]. Additionally, sleep and fatigue showed clinically meaningful improvements. While the effects on orthostatic intolerance were only significant by week four, this suggests potential longer-term benefits of the intervention [5]. Notably, the vast majority (88.2%) of participants reported subjective improvement in their PASC-related symptoms following the treatment. However, two participants experienced no notable change, emphasizing the variability in response and the need for further investigation.

Previous studies have highlighted the presence of dysautonomia in Long COVID patients, with symptoms including orthostatic intolerance, tachycardia, dizziness, and fatigue [5-10]. Our findings align with emerging research demonstrating the efficacy of sympathetic blocks in mitigating symptoms of autonomic dysfunction and chronic pain. For example, a 2021 case series reported sustained improvements in two Long COVID patients following SGB, identifying regional sympathetically mediated dysautonomia as a key contributor to their symptoms [14]. Similarly, research on SGB in patients with complex regional pain syndrome (CRPS) has shown substantial pain relief and improved autonomic function [17]. Research has also demonstrated that SGB is effective in reducing symptoms of post-traumatic stress disorder (PTSD) and anxiety by influencing sympathetic nervous system activity [24]. Given that many Long COVID patients experience neuropsychiatric symptoms such as anxiety and brain fog [1,2], the overlap between PTSD-related sympathetic overactivity and Long COVID-associated dysautonomia suggests that SGB could serve as a valuable intervention in both conditions [6,24]. Our study builds on these insights by providing data from a larger cohort and employing validated outcome measures such as COMPASS-31 and PROMIS-29 to assess changes in autonomic and pain symptoms. The improvements observed across multiple domains, including autonomic dysfunction, pain, sleep, and fatigue, underscore the multi-faceted impact of DSB in addressing PASC symptoms. These results suggest that SGB may help recalibrate abnormal sympathetic signaling, which has been previously hypothesized as a key factor contributing to persistent autonomic symptoms in Long COVID patients [6,9,14].

The role of CBF alterations in Long COVID has been increasingly recognized, with studies suggesting that dysregulated autonomic function contributes to persistent symptoms via impaired vascular regulation [9,10,20,25]. Our findings indicate that SGB may counteract these effects by enhancing CBF and reducing sympathetic-driven vasoconstriction, a mechanism previously documented in studies examining SGB's impact on cerebral perfusion [20]. Additionally, disruptions in CBF have been linked to cognitive impairments such as brain fog and attention deficits, which are frequently reported by Long COVID patients [1,2]. By improving CBF, SGB may help mitigate these symptoms and support cognitive recovery. Furthermore, reduced CBF is often associated with endothelial dysfunction and chronic inflammation, both of which have been implicated in Long COVID pathophysiology [9,10,20,25]. The ability of SGB to modulate these processes suggests a broader potential for vascular stabilization, extending beyond its direct effects on autonomic regulation [10,20]. Future research should investigate whether the improvements in CBF following SGB persist over time and whether repeated procedures can further enhance outcomes for patients with severe Long COVID-related symptoms.

Despite these promising results, several limitations warrant consideration. The open-label, non-randomized design of this study limits the ability to draw definitive conclusions about the efficacy of DSB, as placebo effects and spontaneous recovery cannot be excluded. However, the significant effect sizes and the chronicity of symptoms at enrollment make spontaneous recovery within the short study period less likely. Furthermore, the small sample size and absence of a control group limit how widely our findings can be applied. Future studies should include larger, randomized controlled trials with longer follow-ups to confirm these preliminary results and better understand how DSB works in PASC.

## Conclusions

This study demonstrates that DSBs may be an effective intervention for alleviating sympathetically mediated symptoms in patients with PASC. Participants experienced significant improvements in autonomic dysfunction, pain interference, fatigue, and sleep quality, with many reporting enhanced overall well-being, reduced dizziness, and improved functional mobility. These findings suggest that DSBs have the potential to restore dysregulated sympathetic signaling and provide meaningful symptom relief for individuals suffering from Long COVID.

The observed benefits of DSBs highlight their potential as a minimally invasive treatment option for patients with PASC experiencing persistent autonomic dysfunction and chronic pain. Given the positive outcomes seen in this study, further exploration of DSBs in clinical settings could contribute to expanding treatment options for this patient population, ultimately improving the quality of life for those affected by Long COVID.
